# Bachelor of Nursing students’ HIV and AIDS knowledge in KwaZulu-Natal province: An evaluation study

**DOI:** 10.4102/curationis.v42i1.1928

**Published:** 2019-06-10

**Authors:** Silingene J. Ngcobo, Gugu G. Mchunu

**Affiliations:** 1 School of Nursing and Public Health, Howard College, University of KwaZulu-Natal, Durban, South Africa

**Keywords:** HIV and AIDS education, Bachelor of Nursing students, perceived HIV knowledge, educational programme, programme evaluation

## Abstract

**Background:**

Currently, human immunodeficiency virus and acquired immunodeficiency syndrome (HIV and AIDS) education and training in nursing suffer from various inadequacies and lack any real formalisation in their governance. As a result, Bachelor of Nursing students find themselves challenged in providing effective HIV and AIDS healthcare management, largely because of the deficit in training identified. An HIV and AIDS education intervention programme was introduced at a selected KwaZulu-Natal university to assist in bridging the perceived knowledge gap. This article communicates programme evaluation findings.

**Objectives:**

The aim of this article was to determine levels of HIV knowledge achieved following an HIV education intervention programme.

**Methods:**

A pure, descriptive quantitative research design was employed, using total population sampling (*N* = 133). A modified G3658-11 Collecting Evaluation Data: End-of-Session Questionnaire, developed by the University of Wisconsin–Extension, was administered for data collection.

**Results:**

Females predominated in the study, and most participants were African with 1 to 3 years of education programme exposure. Perceived HIV knowledge increase was evident: pathophysiology (*n* = 93, 70.2%); immunology (*n* = 97, 72.9%); transmission (*n* = 116, 87.5%); diagnosis (*n* = 109, 81.8%); prevention strategies (*n* = 118, 88.4%); staging and monitoring (*n* = 106, 80%); pre- and post-test counselling (*n* = 104, 78%).

**Conclusion:**

Pre- and ongoing in-service HIV and AIDS training can improve perceived HIV knowledge levels for both nursing students and professionals. Mandatory HIV and AIDS healthcare management training is therefore recommended in planning for its effective impartation by nursing educators.

## Introduction

No clear guidelines exist globally outlining specific competencies for nursing students regarding human immunodeficiency virus and acquired immunodeficiency syndrome (HIV and AIDS) training, except for those found to be employed in the USA and Canada (Mill et al. 2014; Relf et al. 2011). Locally, the South African Nursing Council (SANC), which is the profession’s regulating body, has yet to accredit any one HIV and AIDS-related course that provides nursing students with the pertinent knowledge, skills and initial competencies in this sphere of nursing, although externally validated competencies remain regarded as important within the profession (Relf et al. 2004).

It can be argued that much of the HIV and AIDS training currently available is offered only post-diplomate and certificate, with the very limited awarding of these qualifications having become embedded in preservice modules (HEAIDS 2010). Moreover, Relf et al. (2011) attest to a widespread lack of formal HIV and AIDS training and clinical mentoring in courses that are purely didactic both in nature and content, with the training materials available being either outmoded or, culturally speaking, contextually based in only one country’s healthcare system.

Similarly, Mill et al. (2014) concur with the assertion that the present nursing curricula of many institutions have not been effectively revised or updated over time, resulting in them becoming outdated, with inadequate coverage of current HIV and AIDS content, although they are still expected to effectively equip Bachelor of Nursing students with the requisite knowledge and clinical competencies in providing effective HIV and AIDS patient healthcare management.

Such inadequacies in curricula leave Bachelor of Nursing students, enrolled at any accredited nursing training institution providing nursing programmes that culminate in a baccalaureate degree in Nursing, at a serious disadvantage once having completed their studies, by possessing an incomplete or inadequate knowledge of HIV and AIDS healthcare provision.

The literature reviewed for this article reveals that a general and significant lack of HIV and AIDS-related knowledge is apparent amongst nursing graduates (Atul, Ajeet & Kasturwar 2013; Bray Preston et al. 2000; O’Sullivan, Preston & Forti 2000; Pickles et al. 2009; Relf et al. 2011). A similar deduction can be made concerning the statement that poor or inadequate knowledge of HIV and AIDS treatments has led to the provision of substandard clinical care for those patients suffering with them.

Previous research findings, moreover, reveal that the establishment of HIV and AIDS education for nursing students, following the completion of their tertiary qualifications, can result in the provision of far-improved healthcare for people infected with HIV or afflicted by AIDS (Paquin & Lambert 2000). As a result of this postulate, an innovative HIV and AIDS education intervention programme was implemented for undergraduate Bachelor of Nursing students at a selected university within South Africa’s KwaZulu-Natal province, with the goal of enhancing and supplementing the impartation of current knowledge regarding HIV and AIDS to nursing students. Consequent to the implementation of the education intervention programme at the selected university, and also of importance to note, is that a fear of contagion from the virus itself has likewise been significantly ameliorated in nursing students by furnishing them with this kind of training (Makhado & Davhana-Maselesele 2016a).

### Problem statement

Contrarily, no effective or accurate evaluations had been undertaken regarding the outcomes of the HIV and AIDS education programme currently under study, 3 years after the programme’s implementation. Programme evaluation is regarded as extremely important, because it concerns the assigning of value to, and adjudication of, how well any educational programme may be succeeding in equipping students with the knowledge they require professionally (Levin-Rozalis 2003). Fitzpatrick, Sanders and Worthen (2017) further emphasise that the one essential goal of such evaluations is to establish a programme’s ‘usage’, because the use of evaluated findings serves to inform future decision-making by all affected stakeholders for such programmes.

## Aim of the study

The study’s aim was to determine what valuable perceived outcomes had been achieved by the implementation of the HIV and AIDS educational programme at the selected university in KwaZulu-Natal (KZN), utilising a formalised and academically credible evaluation process to do so.

## Background

The study described in this article was carried out 3 years after the HIV and AIDS educational programme’s introduction for undergraduate student nurses at the selected university in KZN, by surveying those who were its recipients. Only those students enrolled in their second to fourth years of study were allocated to the programme; first-year nursing students were excluded, because there are generally high attrition rates amongst them (Jeptha 2008; McLachlan 2010; Roos et al. 2016; Wright & Maree 2007), and furthermore they would not have had enough exposure to the programme during the time of evaluation.

Within the historical context of the university’s School of Nursing, limited HIV and AIDS educational activities were previously offered to their undergraduates. This was additionally true, given that the school previously also employed no lecturers with competencies in HIV and AIDS learning, and therefore also had no tutors with the confidence to instruct nursing students in this subject. It is always challenging to teach on a topic with which one is unfamiliar, and the nature of the programme at the university in KZN was such that it was the first of its kind provided at this university for nursing students. The aspects of the programme that were subjected to evaluation are reflected in Table 1. The programme’s evaluation, through an assessment of the study’s findings, was therefore considered imperative for determining the future success of such programmes overall.

**TABLE 1:** HIV and AIDS knowledge gain.

HIV knowledge items (*N* = 133)	Knowledgeable		Not knowledgeable	
	Number (*n*)	Percentage (%)	Number (*n*)	Percentage (%)
Origins and history of HIV and AIDS	84	63.4	49	36.6
HIV epidemiology	91	68.1	42	31.9
HIV pathophysiology	93	70.2	40	29.8
HIV immunology	97	72.9	36	27.1
HIV transmission	116	87.5	17	12.5
HIV screening and diagnosis	109	81.8	24	18.2
Values clarification, risk assessment and risk reduction	92	69.5	41	30.5
HIV prevention strategies	118	88.4	15	11.6
Stages of HIV and stage monitoring	106	80	27	20
Pre- and post-HIV test counselling	104	78	29	28
Sexually transmitted infections	106	80	27	20
Opportunistic infections	108	81.3	25	18.7
Stigma and disclosure, legal and ethical issues, informed consent and confidentiality	99	74.4	34	25.6
HIV and AIDS management	91	68.4	42	31.6
Introduction of antiretroviral treatments	88	66.4	45	33.6
HIV and AIDS care over time: palliative care	72	54.1	61	45.9

HIV, human immunodeficiency virus; AIDS, acquired immunodeficiency syndrome.

## Trends

Despite the significant decline in HIV incidence rates in South Africa (0.48% reported in 2017 compared to 0.85% reported in 2014 [HSRC 2018]), the country still records the highest number of new HIV infections, particularly in the eastern and southern parts of Africa (Mabaso et al. 2018). This places a great demand for training in this area of study on current and future healthcare providers, in that they need to be better capacitated in terms of the scope of their knowledge, as also in other ways, in order to properly manage this rising stubborn epidemic. Nurses, being the backbone of any healthcare system (Coovadia et al. 2009; Roos et al. 2016), must therefore be encouraged to focus more attention on their current knowledge regarding these ailments.

Because it has been generally observed that nursing students are presently provided with an inadequate or incomplete knowledge of HIV and AIDS patient healthcare management in their training, contemporary recommendations from studies that have been conducted in this area emphasise that a revision of nursing curricula, with particular regard to their HIV and AIDS content, is urgently called for (Bektaş & Kulakaç 2007; Mill et al. 2014; Ouzouni & Nakakis 2012).

The impartation of incomplete and erroneous knowledge, particularly concerning approaches to HIV prevention, its modes of transmission and the pathogenicity of the virus, as well as its progressive form in AIDS, is found to be widespread amongst nursing trainees (Bektaş & Kulakaç 2007). An appeal to improve the skills and subsequent competencies of nursing students through the provision of improved HIV training has therefore been made to those involved in the education sector, as well as to other health professionals, to see undergraduate nurses become better enabled in dealing with those coping with HIV and AIDS-related issues as they are currently perceived within clinical practice (Adhikari et al. 2016).

## Research objective

The primary objective of the study was to determine levels of HIV knowledge acquisition perceived by undergraduate nursing students through the implementation of an education intervention programme at a selected university in KZN, South Africa.

## Definition of key concepts

**HIV and AIDS educational programme:** This term relates to the various HIV and AIDS educational activities undertaken for the trainee nursing complement at the selected KZN university, aimed at the effective training and development of undergraduate nursing students in this area of study.

**Evaluation:** The systematic collection of information regarding the activities, characteristics and outcomes of the educational programme studied, in addition to its services, policies and processes, in order to conduct an assessment of the intervention programme and its processes, with a view to improving its effectiveness, and assist in informing any decisions made regarding its future development.

**Nursing students:** Persons undergoing academic training to become qualified and registered nurses.

**Programme participants:** All nursing students surveyed were in their second to fourth years of study and held active university registrations for training in the discipline of nursing during the years 2013–2015.

## Contribution to the field

The study findings contribute to an evaluation of the effectiveness of the nursing training provided by tertiary educational institutions, in that the curricula for all undergraduate programmes dedicated to the training of nurses are suggested for review and reform, with particular regard to the training provided in HIV and AIDS healthcare proficiencies.

Within the nursing profession, graduates gain additional practical day-to-day skills and knowledge, which must be viewed as essential to them in meeting the particular needs of people within South African communities, especially while this country remains at the global epicentre of the HIV and AIDS epidemic (Shisana et al. 2014), as attested to by the findings presented in the 2010 United Nations General Assembly Special Session (UNGASS) report covering this subject (UNGASS 2010). South Africa is, moreover, the country deploying the largest antiretroviral rollout programme in the world (Morna & Dube 2014), and because of this the effective contextual training of nurses in HIV and AIDS-related matters is deemed to be of the utmost importance.

### Literature review

The provision of current information regarding the HIV virus and its resulting syndrome is considered critical for nurses and nursing students, because these individuals play a vital role in providing healthcare management services to people living with HIV and AIDS (PLWHA) and this ensures provision of the highest quality and most effective standard of healthcare (Dharmalingam et al. 2015). Literature on the subject, however, reveals that HIV and AIDS training within undergraduate nursing programmes is in need of very serious revision and augmentation (Ouzouni & Nakakis 2012), and this is considered a result of a general concern that has arisen regarding the low levels of critical HIV knowledge found amongst nursing students, not just in South Africa but also internationally (Bektaş & Kulakaç 2007; Sikand, Fisher & Friedman 1996; Tavoosi et al. 2004; Tung, Ding & Farmer 2008).

The source of this apparently global inadequacy in nursing competencies for dealing with HIV and AIDS patients can be traced back to an overall paucity of courses offered by institutions of higher learning that comprehensively cover sexually transmitted and other infectious diseases within the scope of their curricula (ElKalmi et al. 2015). According to Frain (2017), a steady decline in HIV and AIDS education across multiple schools of nursing has been observed, with undergraduate nursing students being the group most affected by this. Many of them have reported feelings of unpreparedness for caring for PLWHA, even upon approaching graduation, with this lack of reliable HIV and AIDS healthcare knowledge having many undesirable consequences for nursing students as well as for those in their care.

One finding from the studies reviewed, for example, is that some nursing students hold true that infection with HIV can be prevented by the administration of a vaccine, while others believe that a cure is readily available for the virus (Bektaş & Kulakaç 2007). The nursing students in these studies were moreover observed to entertain many misconceptions regarding how HIV is transmitted, including through the sharing of communal ablutions with an HIV-infected person, which it was thought could result in contracting the virus (Abolfotouh et al. 2013; Mahat & Eller 2009). Another study demonstrated that some nursing students agree with the prognosis that HIV-positive individuals should be quarantined (ElKalmi et al. 2015), while a further study established that nursing students may fail to correctly identify the correct HIV and AIDS medication when administering such treatments (Frain 2017).

In order to ensure a positive impact on undergraduate nursing students regarding an improvement in their preparedness for providing healthcare management services to PLWHA, a recommendation for the incorporation of current and topical HIV and AIDS training into nursing curricula has been forwarded. The providers of nursing training are thereby seen as being handed significant responsibilities in ensuring that nursing students are properly equipped with an understanding of the latest and most accurate HIV and AIDS information available (Frain 2017).

Various sources for HIV and AIDS knowledge have been identified for undergraduate nursing students, although the accuracy of the information provided by these sources is problematic. Studies reveal that student nurses obtain their knowledge of HIV and AIDS, and the treatment thereof, from a variety of sources, including the Internet, magazines, their friends, from watching television and also from their high school teachers (Ouzouni & Nakakis 2012). In keeping with this, 77% of the undergraduate nursing students surveyed for the current study also conveyed that they had not been taught anything regarding HIV and AIDS, or the treatment thereof, throughout the extent of their nursing training, with a small percentage of these students even confirming that they had received no information covering HIV and AIDS from their nursing lecturers at all (Dharmalingam et al. 2015; Ouzouni & Nakakis 2012).

## Description of HIV and AIDS educational programme at a selected site

### Context

The Medical Education Partnership Initiative clinical HIV and AIDS programme is an innovative programme. The main goal of the HIV and AIDS programme was to develop and implement appropriate HIV and AIDS activities for the undergraduate nursing curriculum in the discipline of nursing. The broader objective of the programme is to improve and strengthen the HIV and AIDS content in the existing curriculum, thereby improving the quality and quantity of nursing students trained in the clinical management of HIV and AIDS.

The programme was first introduced and implemented amongst second-year nursing students, where the programme focused on preventative and health promotion, which is an aspect of the community nursing science course. The students got to interact with various communities, with HIV and AIDS being one of the commonest health problems encountered in these communities.

The programme was structured in such a way that each session or activity did not last longer than 60 min. On average the duration was 45–60 min per session.

### The inputs

The inputs that needed to be in place before the HIV and AIDS clinical programme could be implemented were as follows:

- the existing HIV and AIDS curriculum content found in the Bachelor of Nursing programme from the discipline of nursing
- infrastructure
- stakeholders
- project goals
- a budget managed by the principal investigators at the medical school
- tutor training guidelines.

The main person involved in delivering the clinical programme was a professional nurse with an additional postgraduate diploma in clinical management of HIV and AIDS.

### The process

There were six innovative programme processes that were implemented:

- *HIV and AIDS case studies* - The HIV and AIDS case studies in the discipline of nursing were developed in 2011, targeting each year of study, from first to forth year levels. The case studies are reviewed and updated annually.
- *The Nurse Initiated Management of Antiretroviral Therapy (NIMART) training* - is a National Department of Health-accredited training programme that is aimed at capacitating nurses with the skills to initiate HIV-infected patients on antiretroviral therapy. It was offered to all the final-year Bachelor of Nursing students.
- *HIV and AIDS clinical competencies* - The clinical competences have been developed specifically focusing on HIV and AIDS. A total of 20 (Pretoria) HIV and AIDS-orientated clinical competencies have been developed.
  Clinical mentorship - The role of mentors in clinical learning has been described as one that reinforces the correlation of theory to practice.
- *Clinical mentorship* - The role of mentors in clinical learning has been described as one that reinforces the correlation of theory to practice.
- *The HIV morning sessions* - These activities include lectures, group discussions, clinical skills demonstration, an HIV workshop (1 week), use of electronic and visual media, as well as clinical support through mentoring. Students are also given an opportunity to practise any skill that they want to after each session, and such skills can be practised many times, until full mastery of the skill has been achieved by the students. These sessions take place twice a week.
- *The HIV resource room* - The HIV resource room was developed within the nursing clinical skills laboratory. This room contains all relevant HIV and AIDS educational and training materials, in electronic form or in hard copy, as well as equipment and models. The room is accessible to students at all times.

#### Research design

A purely descriptive, quantitative research design model was identified as best for use in establishing the levels of HIV and AIDS knowledge obtained by undergraduate nursing students during the educational intervention programme for HIV and AIDS learning conducted at the selected university in KZN.

A descriptive model was therefore chosen for this study’s research design. It was used to quantify and describe the levels of HIV and AIDS training obtained by study participants through the educational intervention programme discussed herein, which is in keeping with the definition posited by Burns and Grove (2010) describing the elements comprising a descriptive study.

#### Materials

The study comprised 133 of the 237 Bachelor of Nursing students. However, 164 students were targeted, because they were in their second to fourth years of study and had enrolled for HIV educational programme at the selected university in KZN. Total population sampling was employed, with all undergraduate nursing students who had participated in the HIV and AIDS educational programme. Total inclusion of all educational programme recipients in the sample process was considered appropriate for the study, because the literature in this regard indicates that a study population should, where possible, be considered in total, particularly where the size of that population is relatively small, that is, comprising 200 people or less (Christensen et al. 2011). Although a total study population of 164 was targeted, only 133 students agreed to participate in the research conducted, pursuant on their right to refuse to volunteer for, or participate in, this study.

#### Data collection method and procedure

The modified G3658-11 Collecting Evaluation Data: End-of-Session Questionnaire, developed by the University of Wisconsin–Extension, was utilised as adapted for data collection from the study’s population group (Taylor-Powell & Renner 2009). The self-administered questionnaire contained eight sections: Section A: Demographic Data; Section B: Perceived HIV and AIDS Knowledge Gained; Section C: Specific HIV and AIDS Clinical Skills Acquired; Section D: Changes in Attitudes and Beliefs towards HIV and AIDS; Section E: Motivation and Confidence Levels Regarding HIV and AIDS; Section F: Perceptions about Programme Activities; Section G: Perceived Programme Benefits; and Section H: Perceived Limitations of the HIV and AIDS Education Course.

The achievement of this article’s stated objective focuses mainly on the responses obtained to the questions provided for the students surveyed in Section B: Perceived HIV and AIDS Knowledge Gained of the data collection instrument, which was found to be reliable by the application of a Cronbach’s alpha test, resulting in a score of 0.865. The data collection process was done in November 2015 and took place over a divided 2-day period. The self-administered questionnaire was distributed to all targeted undergraduate nursing students and was supported by the aid supplied by two research assistants. The time required to complete the questionnaire by study participants was between 25 and 45 min.

#### Data analysis

Perceived knowledge uptake by the undergraduate nursing students participating in the education programme was measured through a five-point Likert scale, where they were asked as study participants to rate the HIV and AIDS theoretical knowledge and clinical skills understanding that they acquired through participating in the educational intervention programme. Participants’ responses were limited to ‘understanding very well’, ‘somewhat understand’, ‘unsure’, ‘somewhat don’t understand’, and ‘not understanding at all’.

A quantitative framework for analysis of the data collected for the study was established using the Statistical Package for Social Sciences, version 23 for Microsoft Windows, which was also employed in analysing the data obtained from the study instrument. A descriptive statistical analysis of the research data’s means, mode, standard deviation, frequencies and percentages was thence undertaken.

Participants’ responses were thus positioned into two categories, ‘understanding’ and ‘lack of understanding’, and within the context of this evaluation study it was established, from the types of responses provided by the participants, whether they had indeed understood the various topics covered by the educational programme or not. The participants were thereafter categorised into either those who had responded positively as being ‘knowledgeable’ or those who had responded negatively, who were categorised as being ‘unknowledgeable’.

#### Context of the study

Research for this study was undertaken at a selected university in the province of KZN in South Africa, where three universities are situated offering Bachelor of Nursing degrees. This qualification is required for undergraduate learners to be recognised as registered nurses by the SANC following completion of their studies.

### Ethical considerations

Ethical approval was obtained from the departmental and institutional ethics committees for the School of Nursing at the university selected to conduct the education intervention programme, with the reference number BE373/15 assigned.

Prior to the collection of data, full written permission granting approval for evaluative research to be undertaken at the university in support of this study was obtained from the university’s dean and the head of the School for Nursing and Public Health. The university’s registrar provided the additional gatekeeper approval required for the study to commence.

Study participants were informed that no direct benefit or harm was anticipated from participation in the study (Fain 2017), which was being conducted for purely academic purposes. The students were recruited to participate by two research assistants on a voluntary basis, during class sessions. Informed written consent was obtained from all students volunteering to participate in the study. Students were informed that they could withdraw their participation at any time they wished and that would not affect the study progress (Holloway & Galvin 2016). Data obtained from the research performed for the study was stored in a restricted area, according to the institution in question’s data protection and access policies, and no student name was requested in, or appeared on, the questionnaire administered.

## Results

The majority of the nursing students participating in the study were female (*n* = 110; 82.7%); of African origin (*n* = 109; 81.9%); born in South Africa (*n* = 127; 95.5%); and were in their second, third or fourth years of undergraduate study. A significant portion of the students (*n* = 52; 39.1%) had only 1 year of exposure to the educational programme, followed by those with 3 years of exposure (*n* = 47; 35.3%) and those (*n* = 34; 25.6%) with 2 years of programme exposure, as reflected in Table 2.

**TABLE 2:** Demographic profile of participants.

Variable	Frequency (*N* = 133)	Percentage (%)
**Gender**		
Male	23	17.3
Female	110	82.7
**Race**		
African	109	81.9
Mixed race	3	2.3
Indian	15	11.4
White	5	3.8
**Nationality**		
South African	127	95.5
Other	6	4.5
**Year of study**		
2nd level	52	39.1
3rd level	34	25.6
4th level	47	35.3
**Length of HIV and AIDS programme exposure**		
1 year	52	39.1
2 years	34	25.6
3 years	47	35.3

HIV, human immunodeficiency virus; AIDS, acquired immunodeficiency syndrome.

**FIGURE 1:**
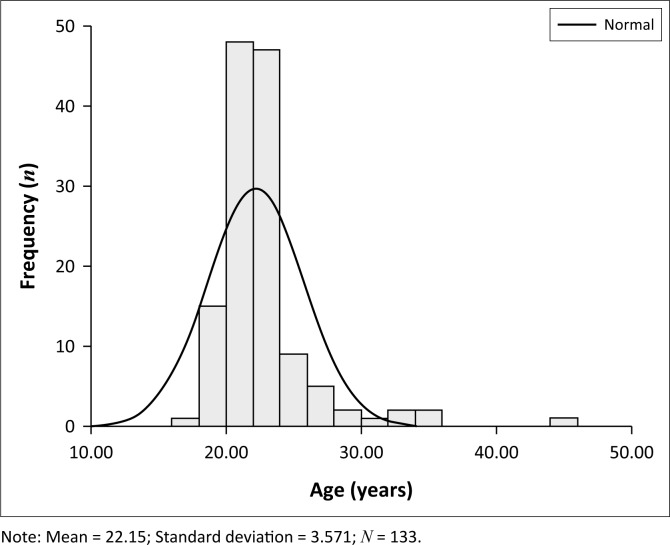
Study population age distribution graph.

The age of study participants ranged from between 17 and 45 years of age. The mean age of participants was 22.2 years (*SD* = 3.6), with a median of 22 years and a mode of 20 years, as reflected in Figure 1. The ages of participants were normatively distributed, because the ages of most of the participants fell close to the mean age for the study population.

Evident from the questionnaire responses obtained from the Bachelor of Nursing students was that they perceived they had indeed gained significant clinical knowledge of HIV and AIDS, and their treatment, from participating in the education intervention programme offered. The students self-reported that they had gained HIV knowledge, especially regarding the following topics: HIV pathophysiology (*n* = 93, 70.2%); immunology and HIV (*n* = 97, 72.9%); HIV transmission (*n* = 116, 87.5%); HIV screening and diagnosis (*n* = 109, 81.8%); HIV-prevention strategies (*n* = 118, 88.4%), stages of HIV and stage monitoring (*n* = 106, 80%); pre- and post-HIV test counselling (*n* = 104, 78%); sexually transmitted infections (*n* = 106, 79.4%); opportunistic infections (*n* = 108, 81.3%); and stigma, disclosure, legal and ethical issues, informed consent and confidentiality (*n* = 99, 74.4%). Overall perceived knowledge of HIV and AIDS obtained from the programme was indicated by the highest score obtained from respondents who indicated being knowledgeable at 87.5% (*n* = 116) and the lowest score of 54.1% (*n* = 72), with the mean average score for this gain being 74.4% (*n* = 99).

## Discussion

Evident from the study findings is that the majority of participating nursing undergraduate students, across all levels of study, self-reported that they gained considerable HIV and AIDS-related knowledge in having become programme recipients. The exposure of Bachelor of Nursing students to HIV and AIDS programmes, such as the one conducted at the selected university in KZN, can therefore be regarded as being of significant importance to both the student nurses and patients within their care, because it orientates and grounds nursing students in their professional practice by providing them with a full and effective knowledge of HIV and AIDS healthcare management, which can continue to be drawn upon in rendering service to patients for the remainder of their postgraduate professional lives.

Graduates should take advantage of all possible opportunities, such as that provided by the educational intervention programme in question, to become better equipped with the most current clinical HIV and AIDS information available, and this can be seen as an imperative, because nurses have always been reported on as being at the forefront of HIV and AIDS healthcare management, from the time the very first case of the epidemic was reported onwards (Makhado & Davhana-Maselesele 2016b; Williams et al. 2006). The general enabling of the acquisition of knowledge by postgraduate nursing students communicates that measures are constantly being planned and implemented to bridge the globally reported gap found to exist in nursing curricula that include HIV and AIDS as a subject; they can be seen as a response to the inadequacies that are seen as present, particularly in the HIV and AIDS course content until recently delivered to undergraduate nursing students (Mill et al. 2014).

A study recently conducted in South Africa reiterates that nursing education with regard to HIV and AIDS still remains in urgent need of the adoption of an improved approach in order for nurses to become better capacitated and supported in their role as HIV and AIDS patient caregivers (Makhado & Davhana-Maselesele 2016a). Such ongoing reports found in the literature reviewed for this study reveal that a lack of adequate HIV and AIDS education and knowledge is both linked with, and identified as, the major cause of negative attitudes, such as fear, anxiety and a reluctance to care for HIV and AIDS-infected individuals, by those providing nursing care to them within the medical service profession (Bray Preston et al. 2000; Jeptha 2008; O’Sullivan et al. 2000). Makhado and Davhana-Maselesele (2016a) additionally establish that negative attitudes, such as the fear of caring for and treating HIV and AIDS patients, can be eliminated by providing nursing students with relevant and appropriate HIV and AIDS training.

This study’s findings are therefore also anticipated to restore hope to HIV and AIDS sufferers in this regard; moreover, they are seen as extremely encouraging, because all of the students surveyed for the study quite evidently experienced significant gains in their knowledge of HIV and AIDS by participating in the educational programme provided for them, which is expected to translate into a higher quality of clinical HIV and AIDS healthcare being rendered to such patients by nursing professionals overall.

In South Africa, a majority of nursing students currently graduate without being in possession of the essential HIV and AIDS training required to effectively perform their work as professional nurses. Only following graduation, and then only where their respective employers deploy them to receive HIV and AIDS training interventions, generally in the form of in-service instruction on the subject, are nursing professionals allowed the opportunity to gain the requisite clinical competencies that will allow them to become effective in their delivery of the appropriate HIV and AIDS healthcare management services required in the field. Although some studies show that in-service HIV and AIDS training can be highly effective (Okpala et al. 2017), it is considered as being of greater benefit to HIV and AIDS patients, who are the ultimate recipients of such overall improvements in healthcare management service delivery that stem from effective preservice training provided to undergraduate nursing students, as demonstrated within the context of this study.

Employers, students and healthcare recipients may all reap enormous benefits from the preservice training indicated here, because employers do not suffer any significant financial deficit, or loss in time devoted to productivity, by its provision, with there concomitantly being no need for additional employee training or other possible staff replacement costs that employers otherwise might incur.

Students can likewise benefit on both a personal and professional level from the introduction of such learning interventions. Especially on a personal level, all information gained from such programmes can be applied in their daily professional lives, which is important because infection with HIV recognises no boundaries.

The study demonstrates that many of the students surveyed for this study also fall into the predefined category for high-risk groups where incidences of infection with HIV are found to be highest. In South Africa, surveys conducted to date have shown that incidences of HIV are highest amongst females within the population who are between 15 and 24 years of age (Shisana et al. 2014).

An additional advantage of receiving preservice training in HIV and AIDS healthcare management for undergraduate nursing students is that they can thereby exert an extended positive influence within the healthcare system at its various service delivery levels. Such a positive influence is seen as being a major advantage of the provision of such training, as opposed to that received by an older generation of postgraduate learners, who are, as a group, reported to be resistant to change or are perceived as failing to embrace any new knowledge with which they are presented, because they wish to continue to operate in the professional manner that they have become accustomed to and comfortable with over time (Hader & Officer 2013). Finally, healthcare recipients (who moreover in this case are PLWHA) represent a population whose members are the primary beneficiaries of the healthcare management of their conditions; they are best enabled by the type of learning described in this study and therefore would experience the greatest additional benefit from the provision of such learning. They are also better advantaged by the delivery of healthcare management being more expert in its undertakings, through the more effective provision of its services, which are thereby made more appropriate in meeting the particular and difficult needs of such people.

The generally positive results obtained from this study indicate that the level of professional confidence for Bachelor of Nursing students is perceptibly boosted by the provision of training intervention programmes like the one under study conducted at the selected KZN university. This has had the additional outcome of promoting their ability to operate more independently with regard to their role as primary HIV and AIDS healthcare givers.

Such attributes are considered desirable for all students to acquire throughout the course of their journey towards both academic and professional development. In the South African context, such attributes are of extraordinary importance, because of the nursing-led healthcare delivery system that has been adopted and promoted as the country’s primary healthcare model.

Comprehensive preservice training can thus conclusively be seen to have an overwhelmingly positive effect by instilling confidence in future healthcare system nursing professionals. Additionally, student leadership qualities can be better capacitated through the implementation of such topical educational intervention programmes as investigated here, because undergraduate nurses are thereby equipped to lead by example in their delivery of clinical HIV and AIDS healthcare management for such patients. Such programmes enable them to teach, guide and supervise those existing postgraduate qualification holders never exposed to the full scope of such learning in the achievement of their higher educational qualifications.

The possession of medical information that is accurate and current empowers the nursing graduates receiving it to provide just such guidance and tuition, especially to those professional nurses who have obtained their qualifications with little or no exposure to the present body of knowledge pertaining to HIV and AIDS healthcare management. Those thus exposed are thereby also empowered to become ‘game changers’ within the field of nursing service delivery. In addition to Paquin and Lambert (2000), Makhado and Davhana-Maselesele (2016a) also confirm in their study findings that the successful provision of improved HIV and AIDS healthcare management services can indeed realistically be achieved through access being allowed to current and appropriate information regarding this area of clinical knowledge for undergraduate student nurses during their undergraduate training.

Educators for the nursing profession consequently have the additional responsibility of ensuring that sufficient HIV and AIDS knowledge is imparted to all Bachelor of Nursing students who pass through their hands in undergoing such learning. Furthermore, they hold the obligation to review and revise their nursing curricula in order to implement adjustments in the learning content they provide, in order for such knowledge amongst both undergraduate and postgraduate nursing trainees to remain both current and topical.

The adoption of a process of review and revision regarding the teaching and learning of the nursing skills that training institutions offer will, moreover, more effectively enable their graduates to provide secure, valuable and compassionate healthcare management services to HIV and AIDS patients, particularly by ensuring that it thereby remains of a sustainably high quality (Pickles et al. 2012). Just as undertaken by the educators at the selected educational institution, this meeting of a perceived challenge in their undergraduate training is considered to be crucial for nursing educators on the whole, because student nurses may lack both adequate basic education and opportunities to improve on their training with regard to obtaining adequate knowledge regarding HIV and AIDS patient healthcare management subsequent to graduation (Mill et al. 2014).

It can moreover be argued that the reason why responsibility should fall to nursing educators to provide such opportunities in student learning is because they may not have been the recipients of any particular or specialised training regarding the delivery of HIV and AIDS healthcare management services themselves. In their defence, therefore, educators can only teach what they have been taught and are also only properly equipped to teach what they already know.

The overall results for this study show that Bachelor of Nursing students did indeed gain significant perceived new knowledge regarding HIV and AIDS patient healthcare service delivery, subsequent to exposure to the education intervention programme instituted at the selected university in KZN, as described in this article.

Improvements in knowledge regarding HIV transmission, for instance, rated highly, with 87.5% of participating students surveyed affirmatively reported that they were now highly familiar with the subject.

Good and sound knowledge in the provision of HIV and AIDS healthcare management services by nursing professionals is therefore seen as being of enormous significance, because those involved in the nursing profession must deal, on an ongoing basis, with the daily preservation of human life. According to Babikian et al. (2004), comprehensive knowledge regarding the possible routes for HIV transmission is not only critical in decreasing infection but also essential in dispelling many of the infection’s persistent myths, and partial knowledge regarding clinical treatment and management of the virus may actually serve to further promote the risk of such infections occurring.

The HIV and AIDS knowledge obtained by students in the learning intervention studied here can also be utilised in the personal lives of nursing students, because information of this kind is highly applicable to them and may enable them to better appreciate and avoid the risk of their own infection with HIV.

This is entirely pertinent because a majority of the students surveyed for this study fall within the vulnerable high-risk age-group category for infection (females between 15 and 24 years of age), where the incidence of HIV is documented as being greatest (Shisana et al. 2014). According to Connelly et al. (2007), Keller et al. (2009) and George, Quinlan and Reardon (2009), it is estimated that one in every seven South African nurses and student nurses are tested positive for HIV infection.

Various studies have been conducted to evaluate the availability of knowledge regarding HIV transmission and prevention for undergraduate nursing students. In reviewing them for this study most are seen to conclude in their findings that nursing students and graduates frequently lack sufficient knowledge for dealing with the transmission and prevention of HIV, which may, in addition, be accompanied by a further problem: the fear of contracting the virus themselves. This is generally seen as a result of an identified lack occurring in the training provided to them for qualification. Included amongst these studies are those having taken place in countries such as Uganda (Mungherera et al. 1997; Walusimbi & Okonsky 2004), Nigeria (Adelekan et al. 1995; Oyeyemi, Oyeyemi & Bello 2006), Tanzania (Kohi & Horrocks 1994), Cameroon (Mbanya et al. 2001) and also South Africa (Mulaudzi, Pengpid & Peltzer 2011).

Some research findings, moreover, show direct similarities with the findings regarding the stated outcomes for the education intervention programme investigated during the study presented in this article, whereby: they postulate that an improved knowledge of HIV and AIDS healthcare management may well be achievable as a result of learning interventions initiated by healthcare training providers. These include similar studies conducted by Taher and Abdelhai (2011) in Cairo and also by Ibrahim et al. (2012), who focus on Malaysian nursing students as their target population.

### Practical implications

This study has implications for nursing education, whereby: planned HIV and AIDS healthcare management programmes yield increased knowledge attained by students, and these will have an impact on the effective clinical practice of nursing.

## Limitations of the study

The study was conducted to investigate the course outcomes for a uniquely innovative HIV and AIDS training programme at only one selected university within the South African province of KwaZulu-Natal, and its findings are therefore not generalisable to other such institutions or programmes. In addition, the variables identified for this study are presented descriptively, with no correlation, either positive or negative, between variables being posited.

## Recommendations

Based on the study findings, the following recommendations can be made:

- There is a need to ensure that preservice HIV training is made mandatory for all nursing students across the country, irrespective of the training already provided for them at either college or university level.
- Nursing educators should undergo mandatory HIV and AIDS training in order to effectively impart knowledge of this subject to their nursing students.
- Enhanced HIV training programmes should be provided for postgraduate students, with similar research being conducted on them to subsequently determine their outcomes.
- Replicas of this study should be conducted once similar training intervention programmes for the purposes of upscaling HIV and AIDS training are established at other institutions of learning, and such studies can additionally be comparative and correlative in their implementation of research design methodology.

## Conclusion

The outcomes for this study provide sufficient evidence that more Bachelor of Nursing students at a selected KZN university perceived that they were in greater possession of added accurate and comprehensive HIV and AIDS healthcare management service knowledge as a result of the innovative HIV and AIDS training programme aimed at enhancing HIV-related curriculum content. The majority of the nurses surveyed may now more confidently render appropriate HIV and AIDS patient healthcare management services to sufferers of the virus and its accompanying syndrome. In doing so, these nurses may also enjoy a greater degree of independence, because of the capacities now invested in them by access to the provision of the programme for imparting contextually relevant knowledge concerning HIV and AIDS.

It is imperative that those tasked with curriculum development for nursing training within the South African Health Ministry, for the nursing profession’s regulating body and at institutions of higher learning, do full justice to their undergraduate students by planning curricula in line with the needs of the country’s healthcare-recipient population. The understanding and treatment of HIV and AIDS are a priority, especially within the South African context, and substantial emphasis, education and training should therefore be focused on them.

It is further evident from the results obtained by this study that education intervention programmes may yield highly positive outcomes, for both nursing graduates and HIV and AIDS patients, such as those already described in this article, where preservice training can be perceived to have far greater benefits than in-service training. The provision of training interventions that deal specifically with the imparting of HIV and AIDS healthcare management skills for delivery to the nursing profession are therefore seen as essential in ensuring that competent nursing postgraduates, who are effective in responding to the country’s needs with regard to HIV and AIDS healthcare management provision, are produced by the training institutions dedicated to this. It is hoped that such a cadre of nursing graduates will be better enabled to independently deal with providing the means for controlling, managing and preventing any complications that may possibly arise as a consequence of the HIV and AIDS epidemic, because they will be qualified for the provision of service in their field by being far more adequately and appropriately equipped.
